# Amniotic fluid neutrophil gelatinase-associated lipocalin and L-type fatty acid-binding protein levels in predicting long-term prognosis in fetal growth restriction and preterm birth: a preliminary study

**DOI:** 10.3389/fped.2025.1712312

**Published:** 2026-01-06

**Authors:** Daisuke Katsura, Shunichiro Tsuji, Shinsuke Tokoro, Ayako Inatomi, Nobuyuki Kita, Takashi Murakami

**Affiliations:** Department of Obstetrics and Gynecology, Shiga University of Medical Science Hospital, Otsu, Japan

**Keywords:** amniotic fluid, infant mortality, long-term prognosis, l-type fatty acid-binding protein, neonatal mortality, neutrophil gelatinase-associated lipocalin

## Abstract

**Introduction:**

The fetal intrauterine environment, including inflammation and fetal hypoxia, influences both short- and long-term prognoses. Urinary neutrophil gelatinase-associated lipocalin (NGAL) and L-type fatty acid-binding protein (L-FABP) levels are associated with inflammation and organ hypoperfusion, respectively. In this study, we evaluated the association between amniotic fluid NGAL and L-FABP levels measured at delivery and long-term outcomes.

**Methods:**

Adverse outcomes were defined as hearing loss, neurodevelopmental impairment, and cerebral palsy. Thirty-one singleton pregnancies were categorized into groups with (AD group, *n* = 10) and without (non-AD group, *n* = 21) adverse outcomes. Patient characteristics, clinical outcomes, and NGAL and L-FABP levels were compared between groups.

**Results:**

Significant differences in the prevalence of fetal blood flow abnormalities (*p* = 0.003) and gestational age at delivery (*p* = 0.004) were observed between groups. NGAL and L-FABP levels were significantly higher in the AD group than in the non-AD group (*p* = 0.015 and *p* = 0.001, respectively). The areas under the curve for NGAL and L-FABP were 0.771 (cut-off: 26,700 µg/gCr) and 0.848 (cut-off: 1,250 µg/gCr), respectively.

**Discussion:**

Amniotic fluid NGAL and L-FABP levels were associated with adverse long-term outcomes, providing preliminary, proof-of-concept evidence of their potential prognostic relevance. Further prospective studies with larger cohorts are needed to validate these findings and clarify their clinical applicability.

## 1 Introduction

Fetal growth restriction (FGR) and preterm birth are associated with both short- and long-term outcomes, including fetal and neonatal mortality, chronic lung disease (CLD), periventricular leukomalacia (PVL), impaired intestinal motility, intellectual disability, motor impairment, and cerebral palsy. These conditions often result from fetal hypoxia in FGR, uterine inflammation in preterm birth, and early gestational age at delivery (1–4). Both short- and long-term prognoses must be considered when managing pregnancy and delivery. Key prognostic factors include gestational age at delivery, birth weight, and the fetal intrauterine environment (2). Assessment of the fetal effects of hypoxia and inflammation serves as an evaluation of the intrauterine environment.

In FGR, fetal condition is typically assessed using ultrasonography and fetal heart rate monitoring as part of the biophysical profile score (BPS) (5), while the timing of delivery is determined based on gestational age, estimated fetal weight, the extent of fetal blood flow abnormalities, and heart rate monitoring (1, 2, 6). Fetal blood flow abnormalities are associated with adverse short- and long-term outcomes, including perinatal mortality, intraventricular hemorrhage (IVH), PVL, necrotizing enterocolitis, and neurodevelopmental impairment (NDI) (7). In cases of uterine inflammation, fetal inflammatory responses may occur even in the absence of maternal signs such as fever, tachycardia, leukocytosis, elevated C-reactive protein levels, or uterine tenderness (8). Therefore, it is important to directly assess the impact of inflammation on the fetus. Although fetal tachycardia, the E/A ratio, velocity time integral, Tei index, cardiac strain imaging, thymus volume, splenic vein flow pattern, and adrenal gland volume on ultrasonography have been proposed as indicators of fetal condition in the context of inflammation, their utility remains limited (9). Findings from fetal ultrasonography and heart rate monitoring may reflect the intrauterine environment but are inherently subjective, with variability introduced by timing and operator judgment. Furthermore, these methods have limited prognostic capability, as monitoring trends over time, rather than relying on a single time point, is essential (6). Thus, there is a need for objective and reliable markers that accurately reflect the fetal intrauterine environment and are associated with prognosis. Amniotic fluid biomarkers may fulfil this need because they originate mainly from fetal urine and directly reflect fetal physiological status.

We previously identified neutrophil gelatinase-associated lipocalin (NGAL) and L-type fatty acid-binding protein (L-FABP) as novel amniotic fluid biomarkers (10–13). Urinary NGAL and L-FABP are established markers for acute kidney injury. NGAL, which correlates with inflammatory markers such as white blood cell count and C-reactive protein levels, exerts protective effects against infection. Conversely, L-FABP, which reflects organ hypoperfusion and oxidative stress, serves an antioxidant function by mitigating lipid peroxidation (10). Specifically, we demonstrated that amniotic fluid NGAL is a useful predictor of fetal inflammatory response syndrome (FIRS), reflecting intrauterine and fetal inflammation (11–13), and that L-FABP is a reliable indicator of fetal hypoxia (11, 12). Additionally, both markers correlate with fetal and neonatal immaturity and may predict short- and long-term outcomes (12, 13). However, these studies primarily focused on short-term neonatal outcomes and did not provide a comprehensive assessment of long-term prognosis.

The present study was designed to build upon previous findings by including children followed up to 2.5 years of age with neurodevelopmental assessments and to clarify the association and preliminary prognostic relevance of amniotic fluid NGAL and L-FABP levels with long-term prognosis.

## 2 Methods

### 2.1 Study design and patients

In our previous studies measuring amniotic fluid NGAL and L-FABP levels at delivery (11–14), we included women with singleton pregnancies who were managed and delivered at the Department of Obstetrics and Gynecology, Shiga University of Medical Science Hospital, Shiga, Japan, between August 2020 and February 2023. In the current srudy, we retrospectively incorporated data from these cohorts, in which children were followed up to 2.5 years of age and underwent standardized neurodevelopmental assessments. Pregnancies complicated by fetal chromosomal abnormalities or major structural anomalies were excluded. Patients with sample turbidity were excluded due to its effect on L-FABP levels (12). All procedures were performed in accordance with the Declaration of Helsinki. This study was approved by the Institutional Review Board of the Shiga University of Medical Science Hospital (approval no. R2022-116). Informed consent was obtained from all patients.

### 2.2 Measurement data

Amniotic fluid samples were obtained during cesarean section using an 18-gauge needle to puncture the membranes prior to rupture, allowing measurement of NGAL and L-FABP levels. Samples were stored at −80°C until analysis. NGAL and L-FABP levels were quantified using a two-step sandwich chemiluminescent enzyme immunoassay kit (SRL, Tokyo, Japan) in accordance with the manufacturer's instructions. Given the influence of urine flow rate on biomarker concentration, creatinine correction was applied. Creatinine correction offers greater diagnostic utility for acute kidney injury than correction by urine flow rate (14). Therefore, similar to previous studies (12, 13), creatinine-corrected values were used for NGAL and L-FABP in amniotic fluid, as accurate assessment of amniotic fluid volume and fetal urine flow rate is difficult.

Maternal characteristics and clinical outcome data were collected, including maternal age, parity, body mass index, gestational age at delivery, birth weight, umbilical arterial pH, and Apgar score <7 at 5 min. Neonatal outcomes included admission to the neonatal intensive care unit, transient tachypnea of the newborn, respiratory distress syndrome (RDS), the need for respiratory support (e.g., nasal directional positive airway pressure and conventional mechanical ventilation), CLD, necrotizing enterocolitis (NEC), IVH, PVL, retinopathy of prematurity, hearing loss, NDI, and cerebral palsy. Hearing loss, NDI, and cerebral palsy were collectively defined as adverse outcomes to assess long-term prognosis. Patients were categorized into groups with (AD group) and without (non-AD group) adverse outcomes for comparative analysis.

FGR was diagnosed as a standard deviation <–1.5 based on the Japanese Society of Ultrasound in Medicine guidelines (15). Fetal blood flow abnormalities were defined as the presence of absent or reversed end-diastolic velocity in the umbilical artery and/or an absent or reversed A-wave in the ductus venosus, each of which is associated with adverse outcomes (2, 16). Intraamniotic infection was defined histologically as chorioamnionitis and funisitis, as these findings serve as diagnostic criteria for FIRS, a condition linked to adverse neonatal events (3). Placental and umbilical cord histopathology was evaluated by qualified pathologists. NDI was defined as a developmental quotient (DQ) <85 at 2.5 years of age. Neurodevelopmental assessments were conducted using either the Enjoji Analytical Developmental Test (17) or the Kyoto Scale of Psychological Development (18), depending on institutional availability and examiner expertise. Both tools are widely used and validated developmental assessment scales in Japan, with DQ scores between 70 and 84 indicating mild developmental delay and scores below 70 indicating moderate or greater delay.

### 2.3 Statistical analyses

Fisher's exact test and the Mann–Whitney U test were used for data comparison. Based on the normality test, variables that met normality assumptions were analyzed using Welch's *t*-test. Receiver operating characteristic (ROC) curves were generated to evaluate the relationship between sensitivity and the false-positive rate (1–specificity) for gestational age at delivery, birth weight, and NGAL and L-FABP levels. The optimal cut-off values were determined using Youden's index.

The areas under the ROC curves and their 95% confidence intervals were calculated for each biomarker, and the areas under the curves (AUCs) were compared using DeLong's test. Statistical analysis was performed using EZR version 1.66 (Saitama Medical Center, Japan) for Windows (19), with *p* < 0.05 considered statistically significant. A power analysis was not conducted, as the study was limited to available data on neurodevelopmental outcomes at 2.5 years of age (19).

## 3 Results

**Figure 1:**
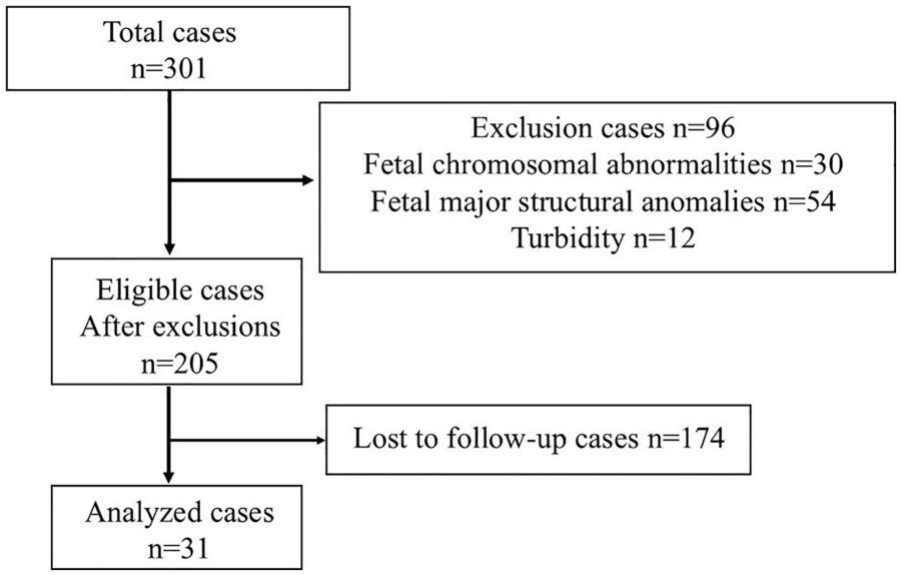
Patient flow diagram.

A total of 301 singleton pregnancies with available amniotic fluid samples were initially screened. Of these, 96 were excluded due to fetal chromosomal abnormalities (*n* = 30), major structural anomalies (*n* = 54), or turbid amniotic fluid (*n* = 12). Among the remaining 205 eligible cases, 174 were lost to follow-up, leaving 31 children with available neurodevelopmental assessments at 2.5 years of age (Figure 1). These 31 cases included 19 cases of FGR and 12 cases of preterm birth. Preterm deliveries that occurred due to fetal indications related to FGR were classified as FGR. These 31 patients were categorized into two groups: 10 in the AD group and 21 in the non-AD group. All patients underwent cesarean section, and amniotic fluid samples were collected via transabdominal approach. Patient characteristics and clinical outcomes are presented in Tables 1, 2, respectively. Significant intergroup differences were noted in the prevalence of fetal blood flow abnormalities, gestational age at delivery, Apgar score <7 at 5 min, incidence of RDS, requirement for respiratory support, and occurrence of CLD and ROP. No cases of gestational diabetes mellitus, NEC, IVH, PVL, or hearing loss were observed. Nine patients presented with NDI, all of whom had DQ scores <85; one had a score <70, indicating moderate delay. One additional child was diagnosed with cerebral palsy. In addition, background characteristics and neonatal outcomes were compared between the FGR (*n* = 19) and preterm birth (*n* = 12) subgroups (Tables 3, 4). No significant differences were observed between the two subgroups.

**Table 1:** Patient characteristics and pregnancy background in the AD and the non-AD groups.

Characteristic	AD-group	Non-AD-group	*p*-value
Number of patients	10	21	
Age (years)^a^	33 (18–42)	32 (24–46)	0.908
Primipara women (%)^b^	30 (3/10)	52.4 (11/21)	0.28
IVF (%)^b^	10 (1/10)	14.3 (3/21)	1
BMI (kg/m^2^)^a^	24.1 (17.5–33.8)	22.9 (18.3–28.5)	0.793
FGR (%)^b^	50 (5/10)	76.2 (16/21)	0.222
Fetal blood flow abnormalities (%)^b^	70 (7/10)	14.3 (3/21)	0.003
Preterm birth	90 (9/10)	57.1 (12/21)	0.106
Intra-amniotic infection (%)^b^	20 (2/10)	9.5 (2/21)	0.577
HDP (%)^b^	40 (4/10)	38.1 (8/21)	1
PE (%)^b^	30 (3/10)	23.8 (5/21)	1

AD, adverse outcomes; IVF, *in vitro* fertilization; BMI, body mass index; FGR, fetal growth restriction; HDP, hypertensive disorder of pregnancy; PE, preeclampsia.

a The Mann–Whitney U-test was used for between-group comparisons, which are presented as median (range), except for age, which was normally distributed and analyzed using Welch's *t*-test.

b Fisher's exact test was used for between-group comparisons.

**Table 2:** Patient clinical outcomes in the AD and non-AD groups.

Characteristic	AD-group	Non-AD-group	*p*-value
Number of patients	10	21	
GA at delivery (weeks)^a^	30.1 (23.6–37.9)	35.6 (26.6–39.6)	0.004
Birth weight (g)^a^	1,150 (551–2,767)	1,612 (818–2,148)	0.78
Umbilical arterial pH^a^	7.323 (7.063–7.474)	7.295 (7.104–7.377)	0.386
Apgar score <7 at 5 min (%)^b^	50 (5/10)	0 (0/21)	<0.001
NICU admission (%)^b^	100 (10/10)	100 (21/21)	1
TTN (%)^b^	10 (1/10)	28.6 (6/21)	0.379
RDS (%)^b^	70 (7/10)	23.8 (5/21)	0.002
Respiratory support (%)^b^	100 (10/10)	52.4 (11/21)	0.011
CLD (%)^b^	50 (5/10)	0 (0/21)	0.001
ROP (%)^b^	30 (3/10)	0 (0/21)	0.026
NI (%)^b^	90 (9/10)	0 (0/21)	<0.001
CP (%)^b^	10 (1/10)	0 (0/21)	0.323

AD, adverse outcomes; GA, gestational age; NICU, neonatal intensive care unit; TTN, transient tachypnea of the newborn; RDS, respiratory distress syndrome; CLD, chronic lung disease; ROP, retinopathy of prematurity; NI, neurodevelopmental impairment; CP, cerebral palsy.

a The Mann–Whitney U-test was used for between-group comparisons, which are presented as median (range), except for Birth weight and Umbilical arterial pH, which were normally distributed and analyzed using Welch's *t*-test.

b Fisher's exact test was used for between-group comparisons.

**Table 3:** Patient characteristics and pregnancy background in the FGR and the PB groups.

Characteristic	FGR-group	PB-group	*p*-value
Number of patients	19	12	
Age (years)^a^	33 (18–46)	32 (26–46)	0.963
Primipara women (%)^b^	47.3 (9/19)	41.6 (5/12)	1
IVF (%)^b^	10.5 (2/19)	16.6 (2/12)	0.63
BMI (kg/m^2^)^a^	23.0 (17.5–28.4)	22.0 (19.6–33.7)	0.604
Fetal blood flow abnormalities (%)^b^	42.1 (8/19)	16.6 (2/12)	0.24
Intra-amniotic infection (%)^b^	5.2 (1/19)	8.3 (1/12)	1
HDP (%)^b^	36.8 (7/19)	41.6 (5/12)	1
PE (%)^b^	36.8 (7/19)	23.8 (1/12)	0.108

FGR, fetal growth restriction; PB, preterm birth; IVF, *in vitro* fertilization; BMI, body mass index; HDP, hypertensive disorder of pregnancy; PE, preeclampsia.

a The Mann–Whitney U-test was used for between-group comparisons, which are presented as median (range), except for age, which was normally distributed and analyzed using Welch's *t*-test.

b Fisher's exact test was used for between-group comparisons.

**Table 4:** Patient clinical outcomes in the FGR and the PB groups.

Characteristic	FGR-group	PB-group	*p*-value
Number of patients	19	12	
GA at delivery (weeks)^a^	36.7 (26.1–39.6)	29.7 (23.6–33.9)	<0.001
Birth weight (g)^a^	1,570 (460–2,767)	1,516 (551–2,598)	0.730
Umbilical arterial pH^a^	7.287 (7.063–7.427)	7.325 (7.121–7.474)	0.361
Apgar score <7 at 5 min (%)^b^	15.7 (3/19)	16.6 (2/12)	1
NICU admission (%)^b^	100 (19/19)	100 (12/12)	1
TTN (%)^b^	26.3 (5/19)	16.6 (2/12)	0.676
RDS (%)^b^	26.3 (5/19)	58.3 (7/12)	0.13
Respiratory support (%)^b^	57.8 (11/19)	83.3 (10/12)	0.24
CLD (%)^b^	10.5 (2/19)	25 (3/12)	0.35
ROP (%)^b^	5.2 (1/19)	16.6 (2/12)	0.543
NI (%)^b^	21 (4/19)	41.6 (5/12)	0.253
CP (%)^b^	5.2 (1/19)	0 (0/12)	1

FGR, fetal growth restriction; PB, preterm birth; GA, gestational age; NICU, neonatal intensive care unit; TTN, transient tachypnea of the newborn; RDS, respiratory distress syndrome; CLD, chronic lung disease; ROP, retinopathy of prematurity; NI, neurodevelopmental impairment; CP, cerebral palsy.

a The Mann–Whitney U-test was used for between-group comparisons, which are presented as median (range), except for Birth weight and Umbilical arterial pH, which was normally distributed and analyzed using Welch's *t*-test. and

b Fisher's exact test was used for between-group comparisons.

**Figure 2:**
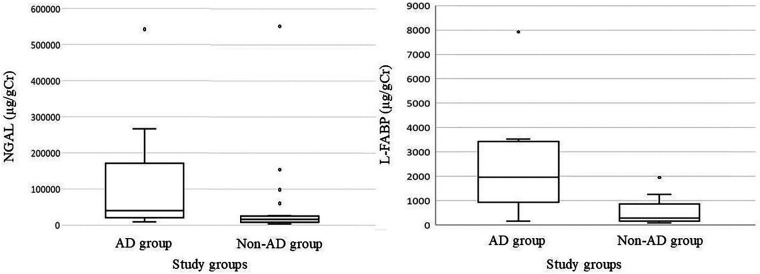
Box-and-whisker plot showing the distribution of amniotic fluid NGAL and L-FABP levels in the AD and non-AD groups. For each plot, the central line represents the median, the box indicates the IQR, and the whiskers represent the minimum and maximum values within a 1.5 × IQR. Individual points outside the whiskers indicate outliers. NGAL, neutrophil gelatinase-associated lipocalin; L-FABP, L-type fatty acid-binding protein; AD, adverse outcomes; IQR, interquartile range.

Associations among NGAL and L-FABP levels and adverse outcomes were examined. As no hearing loss was observed in this cohort, adverse outcomes consisted of NDI and cerebral palsy. NGAL and L-FABP levels were significantly elevated in cases with adverse outcomes (Table 5, Figure 2).

**Table 5:** Comparison of amniotic fluid NGAL and L-FABP levels between the AD group (*n* = 10) and the non-AD group (*n* = 21).

Biomarker	NGAL (µg/gCr)			L-FABP (µg/gCr)		
	AD-group	Non-AD	*p*-value	AD	Non-AD	*p*-value
AD^a^	43,800 (9,100–803,000)	15,900 (3,300–552,000)	0.015	1,955 (154–7,920)	281 (84.4–1,950)	0.001

NGAL, neutrophil gelatinase-associated lipocalin; L-FABP, L-type fatty acid-binding protein; AD, adverse outcomes.

a The Mann–Whitney U-test was used for between-group comparisons, which are presented as median (range).

**Figure 3:**
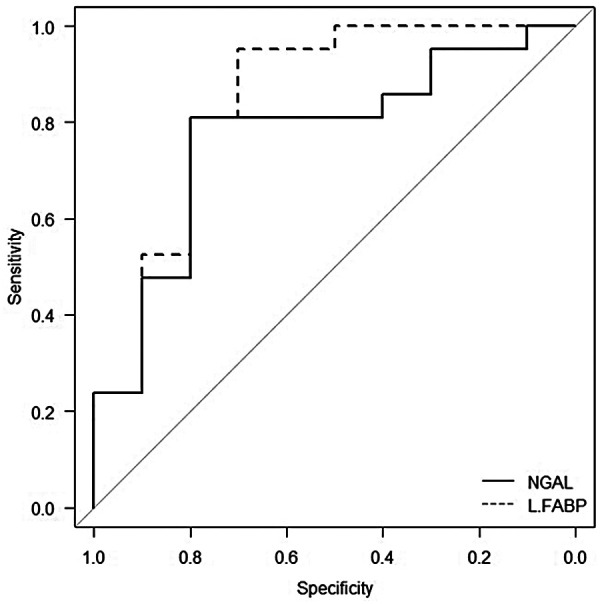
Receiver operating characteristic curves of amniotic fluid NGAL and L-FABP levels overlaid in a single plot for association with adverse outcomes. The *x*-axis represents specificity and the *y*-axis represents sensitivity. The AUCs were 0.771 (95% CI 0.558–0.921) for NGAL and 0.848 (95% CI 0.633–0.976) for L-FABP. NGAL is shown as a solid line, and L-FABP as a dashed line. NGAL, neutrophil gelatinase-associated lipocalin; L-FABP, L-type fatty acid-binding protein; AUC, area under the curve; CI, confidence interval.

We calculated the ROC curves for NGAL and L-FABP levels to evaluate their association with adverse outcomes. For adverse outcomes, the area under the curve and optimal cut-off value for NGAL were 0.771 (95% CI: 0.558–0.921) and 26,700 µg/gCr, respectively, yielding a sensitivity of 0.77 (95% CI: 0.50–1.00) and specificity of 0.76 (95% CI: 0.565–0.941). Those for L-FABP were 0.848 (95% CI: 0.633–0.976) and 1,250 µg/gCr, respectively, yielding a sensitivity of 0.69 (95% CI: 0.417–0.933) and specificity of 0.90 (95% CI: 0.762–1.00). We compared and analyzed the areas under the ROC curves for NGAL and L-FABP levels, which revealed no significant differences between the groups (*p* = 0.599) (Figure 3).

## 4 Discussion

The fetal intrauterine environment reflects short- and long-term prognoses (7). Therefore, the development of prognostic markers that accurately reflect the intrauterine environment is crucial. In this study, we demonstrated that both NGAL and L-FABP may be potential biomarkers associated with adverse long-term prognosis. Our previous studies primarily focused on short-term neonatal outcomes. The present study builds on those findings by including children followed up to 2.5 years of age with neurodevelopmental assessments. This approach provides novel insight into the preliminary prognostic value of these biomarkers beyond the neonatal period.

NGAL, which is expressed in the granules of human neutrophils and several tissues, such as the lungs, liver, and kidneys, exerts protective effects against renal disorders and infections (10). Renal disorders upregulate NGAL expression, and systemic inflammation stimulates NGAL synthesis, resulting in elevated urinary NGAL levels (20, 21). We previously reported that the amniotic fluid NGAL level was a useful predictive factor for FIRS, which is related to infection; its predictive ability was equivalent to that of interleukin-6 and might reflect both maternal and fetal inflammatory conditions and their impact on fetal status (11). L-FABP, expressed in the liver and proximal tubular epithelial cells (10, 22), serves as an indicator of hypoxia resulting from organ hypoperfusion. It binds to highly cytotoxic aldehydes generated during lipid peroxidation following reperfusion, thereby mitigating lipid peroxidative stress (22, 23). We previously reported that the amniotic fluid L-FABP level might be a useful predictor of fetal condition, specifically fetal hypoxia, as well as short- and long-term prognoses (12). Herein, both NGAL and L-FABP levels were significantly associated with long-term prognosis. We speculate that NGAL correlates with inflammation and may therefore be particularly useful for predicting neonatal and infant outcomes related to infection. In this study, although no significant differences in intraamniotic infection were observed between the AD and non-AD groups, NGAL levels were significantly higher in the AD group than in the non-AD group. Because infants in the AD group were delivered at significantly earlier gestational ages than those in the non-AD group, the higher NGAL levels observed in the AD group may reflect the effects of prematurity. By contrast, we speculate that L-FABP levels correlate with hypoperfusion and may be especially useful for predicting outcomes in FGR because they reflect not only prematurity but also fetal condition. In addition, because severe infection can also lead to fetal hypoperfusion, L-FABP might still retain prognostic utility even in cases of infection. Studies on neonates with hypoxic–ischemic encephalopathy have reported that NGAL levels were not associated with the severity of findings on brain MRI findings (24). To our knowledge, no studies have examined the association between L-FABP and neonatal brain imaging findings; however, elevated L-FABP levels have been linked to necrotizing enterocolitis (25). Collectively, these observations suggest that L-FABP may have greater potential as a key biomarker reflecting systemic injury relevant to neurological outcomes, although this remains speculative and requires further investigation. Moreover, immaturity itself directly influences prognosis, and these biomarkers may serve as prognostic indicators, independent of gestational age. Most conventional markers, including maternal blood biomarkers, fetal ultrasonography findings, and fetal heart rate monitoring, can be influenced by maternal conditions and are also susceptible to subjective interpretation by clinicians. By contrast, NGAL and L-FABP are derived directly from fetal urine, providing a more objective and reliable assessment of the fetal intrauterine environment.

Fetal heart rate monitoring and BPS, which are conventional methods for fetal assessment, have false-positive rates of 55%–90% and 40%–50% and false-negative rates of 0.2%–0.65% and 0.07%–0.08%, respectively (26), suggesting that while these methods are useful for screening, they are less reliable for definitive diagnosis. Rather than indicating superiority, our findings suggest that NGAL and L-FABP may provide complementary information to conventional monitoring. Given that measuring amniotic fluid NGAL and L-FABP levels requires invasive amniocentesis (27), its direct application to routine antenatal surveillance is limited. A realistic pathway for current antenatal evaluation would involve biomarker assessment during clinically indicated amniocentesis, such as in patients with suspected intraamniotic infection or in those requiring fetal therapeutic procedures, to avoid additional procedural risk. Once sufficient preliminary data have been accumulated in these high-risk clinical contexts, well-designed prospective studies could be undertaken to determine their role in antenatal decision-making. Going forward, it may be more practical to use these measurements as adjunctive assessments, after initial screening with conventional fetal heart rate monitoring or BPS.

In this study, a significantly earlier gestational age at delivery in the AD group than in the non-AD group resulted in a higher frequency of RDS, respiratory support, CLD and ROP in the AD group. Additionally, the AD group exhibited a higher frequency of blood flow abnormalities and an Apgar score <7 at 5 min than the non-AD group. Since gestational age at delivery, birth weight, fetal blood flow abnormalities, CLD, and an Apgar score <7 at 5 min have been associated with adverse neurological outcomes (2, 28), these results are deemed reasonable. In addition, background characteristics and neonatal outcomes were compared between the FGR and preterm birth subgroups; however, no statistically significant differences were observed, likely due to the small sample size.

This study has several limitations. First, because the number of adverse-outcome cases was extremely small (*n* = 10), neither multivariable regression nor stratified subgroup analyses (e.g., by gestational age or by clinical etiology such as FGR vs. spontaneous preterm birth) could be reliably performed. Consequently, key prognostic factors, including gestational age at delivery and birth weight, could not be adequately adjusted for, and the potential confounding effects of these variables could not be fully addressed. Furthermore, only one child had a DQ score <70 and no cases of hearing loss were identified, making it impossible to use a more clinically specific outcome definition or to analyze NDI and CP separately. This also raises the possibility that the use of DQ <85 may have overestimated clinically meaningful impairment. The wide 95% confidence intervals observed for the AUC, sensitivity, and specificity reflect substantial statistical uncertainty due to the limited sample size. In addition, because this was a retrospective study and amniotic fluid samples were obtained at delivery, only cases with available follow-up data were included, which may have introduced selection bias and limited the external validity and generalizability of the results. Furthermore, future studies should separately evaluate conditions such as infection and FGR, as their distinct pathophysiological mechanisms may affect biomarker performance. Despite these limitations, the present study provides preliminary evidence that amniotic fluid NGAL and L-FABP levels may serve as useful biomarkers of intrauterine compromise and postnatal prognosis. Given the multifactorial nature of adverse perinatal outcomes, reliance on a single biomarker may be insufficient. A combined biomarker approach could improve diagnostic accuracy and may ultimately provide a more precise assessment of fetal condition.

In conclusion, our findings provide preliminary evidence that amniotic fluid NGAL and L-FABP levels are associated with long-term prognosis and may serve as promising biomarkers with potential prognostic relevance. Although measurement requires amniocentesis, these markers could offer objective support in decision-making for high-risk pregnancies.

## Data Availability

The raw data supporting the conclusions of this article will be made available by the authors, without undue reservation.

## References

[1] Baschat AA Viscardi RM Hussey-Gardner B Hashmi N Harman C. Infant neurodevelopment following fetal growth restriction: relationship with antepartum surveillance parameters. Ultrasound Obstet Gynecol. (2009) 33:44–50. 10.1002/uog.6286 19072744

[2] Baschat AA. Neurodevelopment following fetal growth restriction and its relationship with antepartum parameters of placental dysfunction. Ultrasound Obstet Gynecol. (2011) 37:501–14. 10.1002/uog.9008 21520312

[3] Gomez R Romero R Ghezzi F Yoon BH Mazor M Berry SM. The fetal inflammatory response syndrome. Am J Obstet Gynecol. (1998) 179:194–202. 10.1016/S0002-9378(98)70272-8 9704787

[4] Romero R Gomez R Ghezzi F Yoon BH Mazor M Edwin SS A fetal systemic inflammatory response is followed by the spontaneous onset of preterm parturition. Am J Obstet Gynecol. (1998) 179:186–93. 10.1016/S0002-9378(98)70271-6 9704786

[5] Manning FA. Fetal biophysical profile. Obstet Gynecol Clin North Am. (1999) 26:557–77. 10.1016/S0889-8545(05)70099-1 10587955

[6] Lees CC Marlow N van Wassenaer-Leemhuis A Arabin B Bilardo CM Brezinka C 2 year neurodevelopmental and intermediate perinatal outcomes in infants with very preterm fetal growth restriction (TRUFFLE): a randomised trial. Lancet. (2015) 385:2162–72. 10.1016/S0140-6736(14)62049-3 25747582

[7] Soregaroli M Bonera R Danti L Dinolfo D Taddei F Valcamonico A Prognostic role of umbilical artery Doppler velocimetry in growth-restricted fetuses. J Matern Fetal Neonatal Med. (2002) 11:199–203. 10.1080/jmf.11.3.199.203 12380678

[8] Maki Y Sato Y Furukawa S Sameshima H. Histological severity of maternal and fetal inflammation is correlated with the prevalence of maternal clinical signs. J Obstet Gynaecol Res. (2022) 48:1318–27. 10.1111/jog.15241 35509239

[9] Mastrolia SA Erez O Loverro G Di Naro E Weintraub AY Tirosh D Ultrasonographic approach to diagnosis of fetal inflammatory response syndrome: a tool for at-risk fetuses? Am J Obstet Gynecol. (2016) 215:9–20. 10.1016/j.ajog.2016.01.164 26821337

[10] Asada T Isshiki R Hayase N Sumida M Inokuchi R Noiri E Impact of clinical context on acute kidney injury biomarker performances: differences between neutrophil gelatinase-associated lipocalin and L-type fatty acid-binding protein. Sci Rep. (2016) 6:33077. 10.1038/srep33077 27605390 PMC5015077

[11] Katsura D Tsuji S Hayashi K Tokoro S Hoshiyama T Kita N Amniotic fluid interleukin-6 and neutrophil gelatinase-associated lipocalin for predicting fetal inflammatory response syndrome based on histological chorioamnionitis and funisitis. Taiwan J Obstet Gynecol. (2023) 62:516–20. 10.1016/j.tjog.2023.03.014 37407186

[12] Katsura D Tsuji S Tokoro S Inatomi A Hoshiyama T Kita N Evaluation of amniotic fluid neutrophil gelatinase-associated lipocalin and L-type fatty acid-binding protein levels during pregnancy. Eur J Obstet Gynecol Reprod Biol X. (2024) 21:100269. 10.1016/j.eurox.2023.100269 38125710 PMC10733090

[13] Katsura D Tsuji S Hayashi K Tokoro S Zen R Hoshiyama T Amniotic fluid L-type fatty acid-binding protein in predicting fetal condition. Tohoku J Exp Med. (2021) 254:267–73. 10.1620/tjem.254.267 34421087

[14] Tonomura Y Uehara T Yamamoto E Torii M Matsubara M. Decrease in urinary creatinine in acute kidney injury influences diagnostic value of urinary biomarker-to-creatinine ratio in rats. Toxicology. (2011) 290:241–48. 10.1016/j.tox.2011.10.001 22005293

[15] Shinozuka N. Fetal biometry and fetal weight estimation: JSUM standardization. Ultrasound Rev Obstet Gynecol. (2002) 2:156–61. 10.3109/14722240208500478

[16] Vossbeck S de Camargo OK Grab D Bode H Pohlandt F. Neonatal and neurodevelopmental outcome in infants born before 30 weeks of gestation with absent or reversed end-diastolic flow velocities in the umbilical artery. Eur J Pediatr. (2001) 160:128–34. 10.1007/s004310000680 11271385

[17] Enjoji S Goya C. Enjoji Scales of Infant Analytical Development Test. Tokyo: Keioutusin (1980. (in Japanese).

[18] Society for the Kyoto Scale of Psychological Development Test. Shinpan K Shiki Hattatsu Kensahou 2001 Nenban [the Kyoto Scale of Psychological Development Test 2001]. Kyoto: Nakanishiya Shuppan (2008). (in Japanese)

[19] Kanda Y. Investigation of the freely available easy-to-use software “EZR” for medical statistics. Bone Marrow Transplant. (2013) 48:452–8. 10.1038/bmt.2012.244 23208313 PMC3590441

[20] Mori K Lee HT Rapoport D Drexler IR Foster K Yang J Endocytic delivery of lipocalin-siderophore-iron complex rescues the kidney from ischemia-reperfusion injury. J Clin Invest. (2005) 115:610–21. 10.1172/JCI23056 15711640 PMC548316

[21] Mårtensson J Bellomo R. The rise and fall of NGAL in acute kidney injury. Blood Purif. (2014) 37:304–10. 10.1159/000364937 25170751

[22] Yamamoto T Noiri E Ono Y Doi K Negishi K Kamijo A Renal L-type fatty acid—binding protein in acute ischemic injury. J Am Soc Nephrol. (2007) 18:2894–902. 10.1681/ASN.2007010097 17942962

[23] Derikx JP Poeze M van Bijnen AA Buurman WA Heineman E. Evidence for intestinal and liver epithelial cell injury in the early phase of sepsis. Shock. (2007) 28:544–8. 10.1097/shk.0b013e3180644e32 17607153

[24] Sweetman DU Onwuneme C Watson WR O'Neill A Murphy JF Molloy EJ. Renal function and novel urinary biomarkers in infants with neonatal encephalopathy. Acta Paedistr. (2016) 105:e513–e9. 10.1111/apa.13555 27551944

[25] Coufal S Kokesova A Tlaskalova-Hogenova H Frybova B Snajdauf J Rygl M Urinary I-FABP, L-FABP, TFF-3, and SAA can diagnose and predict the disease course in necrotizing enterocolitis at the early stage of disease. J Immunol Res. (2020) 2020:3074313. 10.1155/2020/3074313 32190704 PMC7072107

[26] Signore C Freeman RK Spong CY. Antenatal testing-a reevaluation: executive summary of a eunice kennedy shriver national institute of child health and human development workshop. Obstet Gynecol. (2009) 113:687–701. 10.1097/AOG.0b013e318197bd8a 19300336 PMC2771454

[27] Kalogiannidis I Prapa S Dagklis T Karkanaki A Petousis S Prapas Y Amniocentesis-related adverse outcomes according to placental location and risk factors for fetal loss after midtrimester amniocentesis. Clin Exp Obstet Gynecol. (2011) 38:239–42. 21995155

[28] Thébaud B Goss KN Laughon M Whitsett JA Abman SH Steinhorn RH Bronchopulmonary dysplasia. Nat Rev Dis Primers. (2019) 5:78. 10.1038/s41572-019-0127-7 31727986 PMC6986462

