# Supplementary figures and images for: Amniotic fluid neutrophil gelatinase-associated lipocalin and L-type fatty acid-binding protein levels in predicting long-term prognosis in fetal growth restriction and preterm birth: a preliminary study
Source: Front Pediatr. 2026 Jan 6;13:1712312. doi: 10.3389/fped.2025.1712312 (PMC12816317; doi:10.3389/fped.2025.1712312)

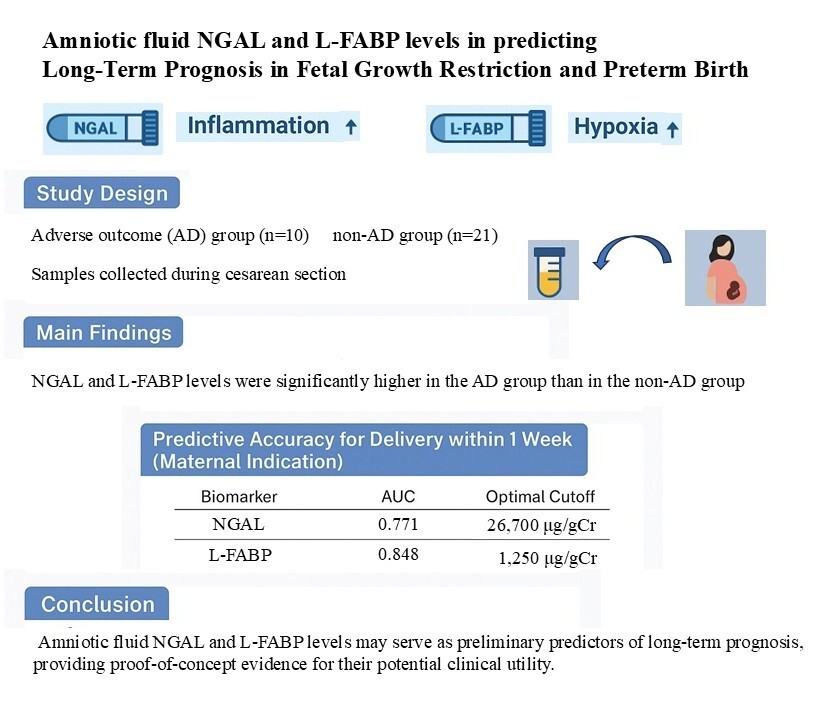

Supplement: Supplementary file 1 [file Image1.jpeg]
